# What is risk in clinical genetics? Designing and piloting tools to evaluate risk in clinical genetics using failure modes and effects analysis

**DOI:** 10.1038/s41431-025-01961-3

**Published:** 2025-10-27

**Authors:** Deborah M. Lambert, Helen Stewart, Mari Bandiola, Marta Bertoli, Dearbhla Butterly, Outi Kuismin, Jukka Moilanen, Gillian Rea, Ioana Streata, Anna Griffin, Vicky McGrath, Deborah Behan, Natasha Coen, Sheila King, Elaine Kilroe, Alana J. Ward, Sally Ann Lynch

**Affiliations:** 1 University College Dublin, Belfield, Dublin, Republic of Ireland; 2 Rotunda Hospital, Dublin, Republic of Ireland; 3 Oxford Centre for Genomic Medicine, ACE building, Headington, Oxford UK; 4 Northern Genetics Service, Central Parkway, Newcastle upon Tyne, UK; 5 Department of Clinical Genetics and Rare Disease Unit, Research Unit of Clinical Medicine, Medical, Research Center Oulu, Oulu University Hospital and University of Oulu, Oulu, Finland; 6 Northern Ireland Regional Genetics Service, Belfast, Northern Ireland UK; 7 Laboratory of Human Genomics, University of Medicine and Pharmacy of Craiova, Regional Centre of Medical Genetics Dolj, Emergency Clinical, County Hospital Craiova, Craiova, Romania; 8 University of Glasgow, School of Medicine, Dentistry and Nursing, Wolfson, Medical School Building, Ave, Glasgow, Scotland UK; 9 Rare Disease Ireland, Dublin, Republic of Ireland; 10 Risk Department, Children’s Health Ireland, Dublin, Republic of Ireland; 11 State Claims Agency, Treasury Dock, North Wall Quay, Dublin, Republic of Ireland; 12 Enterprise Risk Management, Risk Office of the Chief Strategy Officer, Dr Steevens Hospital, Dublin, Republic of Ireland; 13 Department of Clinical Genetics, Children’s Health Ireland, Dublin, Republic of Ireland

**Keywords:** Genetic counselling, Genetic testing

## Abstract

Risk exists throughout medicine. Understanding health system pressure points permits implementation of controls for risk reduction. The literature lacks a systematic approach to risk evaluation in Clinical Genetics. We aimed to develop Clinical Genetics-specific risk assessment tools to prospectively monitor risk. A retrospective review of 115 cases with identified adverse events or near misses in Clinical Genetics in Ireland was used to design a process map to define the steps where risk occurs across the patient journey through clinical genetics. We piloted a clinical audit form using the process map to capture risk event frequency. The draft process map and audit form were trialled (2022–2023) in 5 other European clinical genetics centres for validity and usability, and 2 re-audited in 2024 to assess utility. Using narrative summaries from the case review, we modified the national health risk severity scoring for clinical genetics use. The design of the risk process map, risk frequency audit and severity assessment align with Failure Modes and Effect Analysis methodology. Adverse events occurred in >3% of appointments in 4 of 6 centres (range 0.8–20.3%). High frequency failure modes varied by centre and included consent, sample processing, and patient discussion. Re-audit results reflected interventions introduced since initial audit. We propose these tools provide a standardized approach to discussing systematic risk in clinical genetics, and can be used to prospectively monitor adverse events, allowing controls to be put in place, reducing risk, thereby improving quality of service.

## Introduction

The Irish Health Service Executive (HSE) defines risk as ‘the effect of uncertainty on our objectives. Risk management is therefore about how we manage those uncertainties to give us the best chance of success in meeting our objectives.’ [1]. It is fundamental to proactively manage, anticipate, and respond to risk ensuring safe delivery of care [1–3]. Risk management measures can lead to quality improvement, defined as the combined and unceasing efforts of healthcare professionals, patients, and families to make changes that will lead to better patient outcomes, better experiences of care, and continued development and supported staff [4].

What is risk in clinical genetics? The World Health Organisation describes Clinical Genetics as a preventative specialty [5]. As such, the consequences or impact of any risk event may not be evident for years or generations. Notably, in Clinical Genetics, compared to other specialities, the impact of an adverse event may extend beyond the individual to impact the family [6]. In addition to minor harms, severe harms in Clinical Genetics include a recurrence of an inherited condition that might have been avoided had the family been informed of the risk, the occurrence of a cancer that might have been prevented, or disability due to a delay in medical treatment or dietary restrictions. More recently, misinterpretation of complex genetic test results has resulted in high-profile cases of harm because of interventions that were subsequently shown not to be indicated, e.g., terminations of pregnancy [7] or prophylactic mastectomies for DNA variants that were later interpreted as benign [8]. The risk of severe adverse events has been used as a reason to call for the regulation of genetic counsellors [9]. Tools have been developed to assess quality (good care) in clinical genetics and genetic counselling based on service structures, process of care and health and patient outcomes [10], but these are *quality* and not *risk* (lack of safe care) assessment measures.

Members of our research team have a longstanding interest in risk factors in clinical genetics practice such as staffing and length of wait to appointment [11, 12]. However, these were evaluations of service delivery on practice and did not examine the patient journey. In order to meet our aim of assessing and describing risk across the patient journey through clinical genetics, we needed to determine the most appropriate methodology to do so. With respect to practice, types and magnitude of risks in prenatal diagnosis have been described [13], as has the pathway of clinical referral and first visit in a genetics clinic [14]. In genetics laboratory risk analysis, Claxton et al. [15] describe prospective risk assessment of the laboratory process of genetic variant testing by failure modes and effects analysis (FMEA). Donohue et al. [7] provide a narrative exploration of challenges of genetic test misinterpretation and the resulting clinical impacts captured in a survey of 360 American genetics providers. A literature review of the subject showed few publications related to risk across the patient journey in clinical genetics.

In contrast, Clinical Genetics Laboratories have systematic, dedicated process risk assessment for each laboratory process as part of Quality Management Schemes [16] and ISO compliance such as ISO 15189 [17]. Considering the complexity of genomic testing, including request, reporting, and consent requirements, many laboratories have developed process maps specific to sample processing and reporting as part of Internal Quality Control [18]. Our interest lies in describing the clinical genetics pre- and post-test activity that ‘sandwiches’ the genetics laboratory activity.

Our process of risk assessment tool development best aligns with Failure Modes and Effects Analysis. FMEA differs from hospital clinical risk reporting in that it prospectively examines a pathway to identify risks; while hospital risk reporting retrospectively examines adverse events and/or near-misses to explore the cause and severity of risk the occurred [19]. While both approaches allow for risk identification to mitigate risk leading to service improvements, FMEA can proactively recognize risk, may prevent risk [19] and fits within hospital quality improvement programs [20].

FMEA is well aligned to the aim of our project, as methodology is based on the creation of a risk process map, followed by consideration of risk frequency and severity to define a risk score and identify risks [15, 19–21]. FMEA methodology involves 5 steps [19, 21]:

1. Define the high-risk process.
2. Assemble of a multidisciplinary team including subject experts and risk management experts.
3. Graph or map the process, with each step having a risk of an adverse event or near miss (called a failure mode).
4. Conduct a hazard analysis for each step in the process, by calculating the frequency and the severity (impact) of the failure mode. ‘How often do we see a failure at this step, and how severe is the impact?’.
5. Define action measures to mitigate the most hazardous risks.

FMEA step 4 of hazard analysis [19] dovetails with the Irish HSE Incident Management Framework [22] and Risk Assessment Tool [23] which use a Traffic Light Risk Assessment system of hazard analysis. The Traffic Light risk assessment system uses a risk matrix of frequency and impact to grade risks as green (low-), amber (medium-) or red (high-risk) [23]. This tool can be used both prospectively and in risk review of incidents [24]. In order to align our FMEA analysis with this existing HSE framework for risk analysis, we adopted the Traffic Light Risk Assessment system in step 4 of our FMEA analysis.

The HSE Risk Assessment Tool [23] considers risk impact categories: harm to a person (patient/staff/public), service user experience, business/service disruption, loss of trust/confidence, organizational outcomes/objectives, compliance requirements, financial harm to health service, environmental harm, and health service strategic programme/project harm. Eight of these harms are generic to all specialties, however, “harm to person” can be specialty-specific. The HSE Risk Assessment tool describes ‘harm to person’ in terms of physical injury (no first aid required to loss of life) or psychological damage (no impaired psychosocial functioning to permanent psychosocial impairment). While this aligns well with surgical harms for in-patient use, it does not completely describe the harm severities of clinical genetics being a preventive speciality [5] and the familial nature of clinical genetic harms [6].

This study outlines our creation of a framework of process mapping and hazard analysis to produce tools for risk analysis in clinical genetics.

## Methodology

### Scope

The scope of FMEA analysis was defined as risks to the patient moving through outpatient Clinical Genetics to obtain genetic testing and/or genetic diagnosis, including receiving genetic testing through mainstream (i.e., non-genetic) clinics and subsequently attending Clinical Genetics for interpretation of results. The methodology for process map development is outlined as the framework described by Antonacci et al. [25].

### Research teams

The overall study design was conceived by the core research team and included a Consultant Clinical Geneticist (SAL) and two European Registered Genetic Counsellors (AJW and DL), all of whom had undertaken Clinical Risk training with national risk management agencies, a MSc trained research assistant (DB) and a patient representative, VMcG who as a trained engineer has an understanding of risk processes. AJW is also a PhD molecular genetics scientist with extensive experience in laboratory quality and processes.

External participating Clinical Genetics teams (Oxford (HS and MB), Newcastle (MB2), Oulu (OK and JM), Northern Ireland (GR), and Dolj (IS)) were recruited through the European Reference Network for Rare Malformations (ERN ITHACA) and through the British Society of Genetic Medicine in 2022. The participating Risk experts were recruited by direct contact from the Principal Investigator and included a hospital risk manager (DB2), a member of the Health Service Executive’s Enterprise Risk team (EK) and a senior clinical risk manager and a clinical risk advisor with the State Claims Agency (NC and SK) which advises and assists State authorities with risk management services and resolves third-party claims against State authorities.

**Fig. 1:**
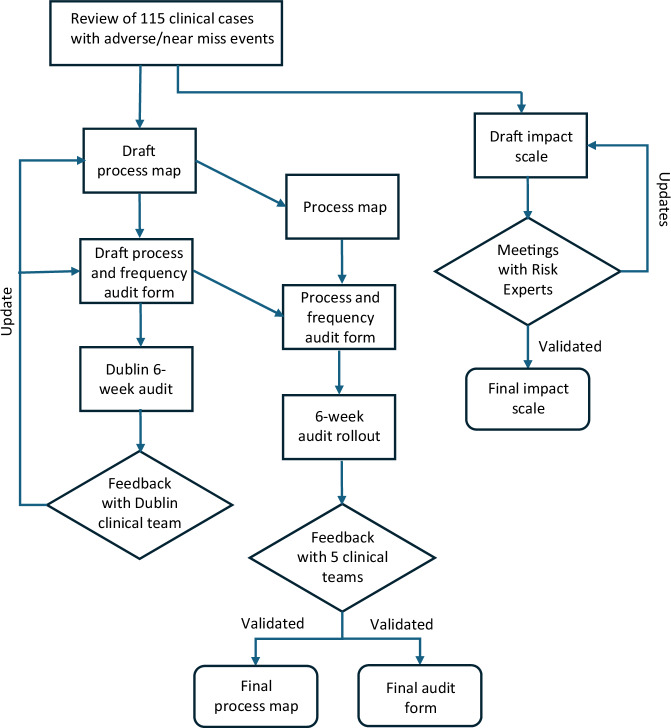
Methodology Flowchart. Development of clinical genetics process map, audit form, and impact scale development from clinical case review of adverse and near-miss events, aligned with FMEA methodology.

See Fig. 1 for an overview of methodology used for FMEA tool development.

### Information sources

1. Clinical genetics charts with risk events. This was a selective, purposeful sampling of genetic charts captured retrospectively by SAL over a 3-year period in Clinical Genetics in Children’s Health Ireland. Charts were identified when they contained adverse events (that had been reported to the Hospital Clinical Risk department, Laboratory reporting risk systems, and/or Data Protection Office for data breaches), or contained near-miss events where significant time had been taken by Clinical Genetics staff to prevent the clinical situation escalating to an adverse event.
2. A concurrent survey of Rare Disease families, which was undertaken as part of this research grant [26] captured the impact of harm on families, confirming the harm that can occur. This informed the development of the impact severity scale.

Data for the 115 genetic charts with risk were captured in Excel. Hospital ID, diagnosis, recurrence risk, waiting time from referral to first appointment, age category of proband (fetal/neonate/child/adult), and speciality of referrer were recorded. Cases were reviewed to determine all proximate cause(s) of the adverse event or near miss. All cases were independently reviewed by 2 or more members of the core research team, each case was discussed amongst the team until consensus on proximate causes of the risk was reached. The harm to the proband or family was recorded as a narrative summary statement.

### Creation of a process map

All proximate causes of risks identified were organized in sequential order to create a 22-step process map of the patient journey through clinical genetics or genetic testing, based on clinical knowledge of the core research team. The process map was plotted in Biorender.

### Creation of a risk frequency audit form

The frequency of missteps (failure modes) could not be established from the purposeful sampling of 115 risk charts, so an audit form for prospective ascertainment was developed. This incorporated the process map, with questions regarding the demographics of the reporter, a broad de-identified disease category, a 1-sentence summary of the nature of the event, and whether the risk occurred in genetics or a mainstream clinic. A 6-week audit period was chosen based on the researchers’ professional estimate of adverse event frequency. The full clinical audit instructions and data collection form can be found in Supplemental Fig. 1.

A prospective audit was undertaken at Clinical Genetics, CHI-Crumlin, Dublin, to trial the audit form’s usability and assess the frequency of events. Members of the clinical team completed one audit form for each appointment where an adverse event or near miss was identified. The total number of outpatient clinic appointments within the Centre in the audit period was obtained from the administrative team. At the end of the 6-week period, audit data were compiled in Excel for analysis. Frequency for each step along the map was calculated as the number of events per total number of appointments in the audit period.

Following data analysis for the CHI-Crumlin audit, a feedback meeting was held with the CHI Clinical Genetics team. The research team provided results on the frequency of events observed, and a general discussion about the perception of risk was held. Feedback was sought on the completeness and composition of the process map and the ease of use and wording of the audit form. The research team incorporated the team’s feedback into the process map and audit form.

The audit was rolled out to the 5 participating clinical genetics centres. Characteristics of the participating centres, including catchment, scope of patients included in audit, and type of clinical staff on team, are shown in Table 1. Belfast and Oxford produced an electronic version of the audit form for their own use. Centres provided the number of outpatient genetics appointments given during the audit period. All 5 centres provided Excel spreadsheets of results to the core research team. Feedback meetings were held with each centre upon audit completion to determine redundant or missing steps in the process map. They were also asked if the audit form was simple and time-efficient to complete, and whether a summary of the findings permitted them to reflect on risk within their clinical practice. The frequency of events at each step was calculated as the number of events per the total number of appointments in the audit period.

**Table 1:** Characteristics of auditing centres and audit data captured.

Centre	Service, Catchment	Fetal	Paed	Adult	Cancer	Inpt	Outpt	CG	GC	GN	GP
Dublin	Tertiary hospital, national	✓	✓	✓	✓	×	✓	4	7	0	0
Belfast	Tertiary hospital, national	✓	✓	✓	✓	✓	✓	6	6	0	0
Craiova	Tertiary hospital, regional	✓	✓	✓	✓	×	✓	5	0	3	0
Oulu	University, tertiary hospital, ½ country	✓	✓	✓	✓	×	✓	8	0	6	0
Oxford	Tertiary hospital, regional	×	✓	✓	×	×	✓	13	5		0
Newcastle^a^	Tertiary hospital, regional	✓	✓	✓	✓	✓	✓	6.5	6.9	0	2

Clinical Genetics centres participating in 6-week audit, 2022. Types of patients included in study: fetal=prenatal; paed=paediatric; adult and cancer. Types of appointments: inpt=inpatient; outpt=outpatient. Number of clinical staff on centre team at time of audit: *CG* consultant geneticist, *GC* genetic counsellor, *GN* genetic nurse, *GP* Genomic Practitioner/Associate. ^a^Newcastle staffing figures given as whole-time equivalents.

### Adaptation of impact (Severity) score

In parallel to the development of the process map and audit, the research team engaged with Risk experts in order to understand how adverse events are managed on a national basis and how this could be applied to a clinical genetics’ context. The impact of risk on patients and their families from the 115-case review as well as from the public survey on clinical genetics and genetic testing, was reviewed within the core research team. Generalized and anonymized versions of risks identified in the case review and public survey were discussed with Risk experts. While originally consideration was given to adaptation of all categories of the risk assessment tool [1, 23], it was decided that most categories (such as harm to the environment, financial, reputational harm to the health service) did not differ from other medical specialties. The risk severity levels on the Irish public health service risk assessment tool [1, 23] for the category ‘Harm to person (Service User, Patient, Staff & Public)’ were adapted into a Clinical Genetics-specific tool by the team by considering the individual and family-specific outcomes particular to Clinical Genetics at different severity levels.

## Results

### Tools development

The 115 charts reviewed had a time from referral to appointment ranging from 0 to 39 months. 65/115 charts were related to pregnancies that occurred since referral: consultand requested an expedited appointment because pregnant, consultand declared a pregnancy at appointment, or consultand attended appointment, and pedigree analysis revealed the birth of subsequent children since referral. Of note, 3 pregnant women were affected by an autosomal dominant condition with a significant risk to their offspring. 41/115 had urgent genetic testing in order to resolve a genetic diagnosis to facilitate prenatal testing.

The remaining 50/115 charts were unrelated to pregnancy at the time of appointment –11 of these were cancer genetics. 10/115 charts were regarding recurrence for the death of a fetus or baby with a possible genetic syndrome. A sample of de-identified narrative summaries from the charts is shown in Table 2 – some of the vignettes contain more than one adverse event or near miss.

**Table 2:** Vignettes from cases reviewed.

Risk	De-identified summary
Incorrect risk assessment	Couple given a high recurrence risk of a baby with sporadic congenital malformation by a mainstream clinician. Partner has a vasectomy. Attends genetics and given a low recurrence risk, very upset.
Phenotype not recorded	Patient referral for isolated congenital malformation not accepted into the genetics service. Patient re-referred to genetics years later after extreme but preventable health consequences of their syndromic congenital malformation.
Incorrect information on laboratory requisition	CNV interpretation in baby reported as VUS as neither parental sample had CNV, however mainstream clinician did not declare that maternal blood sample was from the birth mother and not egg donor. Final analysis: egg donor carries CNV, CNV benign.
Incorrect test ordered	Parents of a child with an imprinting disorder were offered prenatal diagnosis for the disorder by mainstream clinician, but offered diagnostic test for incorrect disease mechanism
Lab not informed of specialised test	Fetal medicine team organized prenatal diagnosis for Robertsonian translocation but did not inform lab of translocation or request UPD studies – incomplete analysis, possible false reassurance
Incomplete consent	Trio exome testing for isolated deafness by mainstream clinician returns result of X-linked ACMG secondary finding in mother. Mainstream clinician not aware genes unrelated to deafness tested, does not know what secondary finding is, family not consented adequately for test.
Incorrect sample taken	Array revealed a large possible unbalanced translocation in child, parental samples in lithium heparin requested by testing laboratory to exclude possible balanced translocation in either parent, samples in EDTA sent in error.
Critical sample missed	Couple whose baby was deceased due to a possible underlying genetic disorder. By the time of their appointment, the sample from the deceased baby had been discarded.
Lab processes sample incorrectly (identifiers not checked)	Multiple family members organized private genetic testing via saliva samples, attend public clinical genetics for results interpretation. Lack of identifiers on original samples meant that results for different family members cannot be distinguished; testing must be redone.
Duplicate test	Paediatric patient has second exome test with no additional indication - not detected as sent to different external lab
Incorrect external lab	Pregnant patient whose child had died of a rare autosomal recessive disorder diagnosed by clinical genetics. Regional maternity service subsequently offered antenatal testing without knowing molecular cause or original testing laboratory - were planning to send sample to external cytogenetics laboratory.
Sample undergoes incorrect test	Proband identified as being compound heterozygote for 2 pathogenic variants in a rare disease gene. Same external lab tests parents, returns result that neither is a carrier. Laboratory undertakes review at request of clinical geneticist; incorrect test carried out on parental samples.
Inappropriately worded result	Family-based testing done for X-linked syndromic intellectual disability syndrome. Child’s report states de novo variant identified, but the mother’s reports she is a carrier of same variant. Discrepancy not noticed by referring mainstream clinician.
Result not sent to requesting clinician	Mainstream clinician sends sample to external lab, no genetic diagnosis found. Patient seen in clinical genetics years later, geneticist requests further testing on stored sample in external lab. No results returned to geneticist, all result sent to mainstream clinician, who no longer treats patient.
Clinician does not receive report	Hospital IT system blocks all correspondence and genetic result from external lab to consultant geneticist because of spam filtering
Incorrect comparison of genotype to phenotype	Mosaic karyotype found on amniocentesis in fetus with intrauterine growth retardation. Counselled by obstetrics that this is a fatal fetal anomaly. Baby born small, but well; genetics team explained that phenotype in a mosaic form can be much milder
Incorrect result interpretation	Patient arrived into country requesting interpretation and possible diagnosis for child with significant developmental delay. Exome results from abroad had revealed a heterozygous variant of unknown significance in an Intellectual disability gene which one of the parents (both healthy) also carried. Previous prenatal testing offered abroad resulted in 2 terminations of pregnancy due to presence of the VUS. Review of exome results showed that the heterozygous variant was benign.
Incorrect test/incorrect result interpretation	Couple where man had microdeletion syndrome was offered NIPS screening for microdeletion syndrome by the fetal medicine specialist without consideration of the correct Bayesian prior probability, very low clinical utility
Possibility of result reclassification	Trio exome reported that no clinically significant variants found. Clinical geneticist requests reinterpretation – hypomorphic allele had been filtered out in original analysis but gives significant result if considered.
Sub-optimal result communicated to family	Results of a cancer predisposition gene given, but mainstream clinician did not take into consideration this was an imprinted gene so gave incorrect interpretation
Clinician does not refer appropriately	Child has CNV on array and told there is an abnormality which one ofthe parents carries, family not referred to clinical genetics. Referred later by another mainstream consultant and review of CNV reveals it is benign
Inappropriate waiting time	Couple had second child with recessive skeletal dysplasia in time between referral to clinical genetics and appointment for discussion of recurrence risk.
Ethical issue	Proband underwent diagnostic testing for cancer gene after bone marrow transplant. Transplant not declared to lab. Proband had received transplant from family member, test gave diagnostic result for donor.

**Fig. 2:**
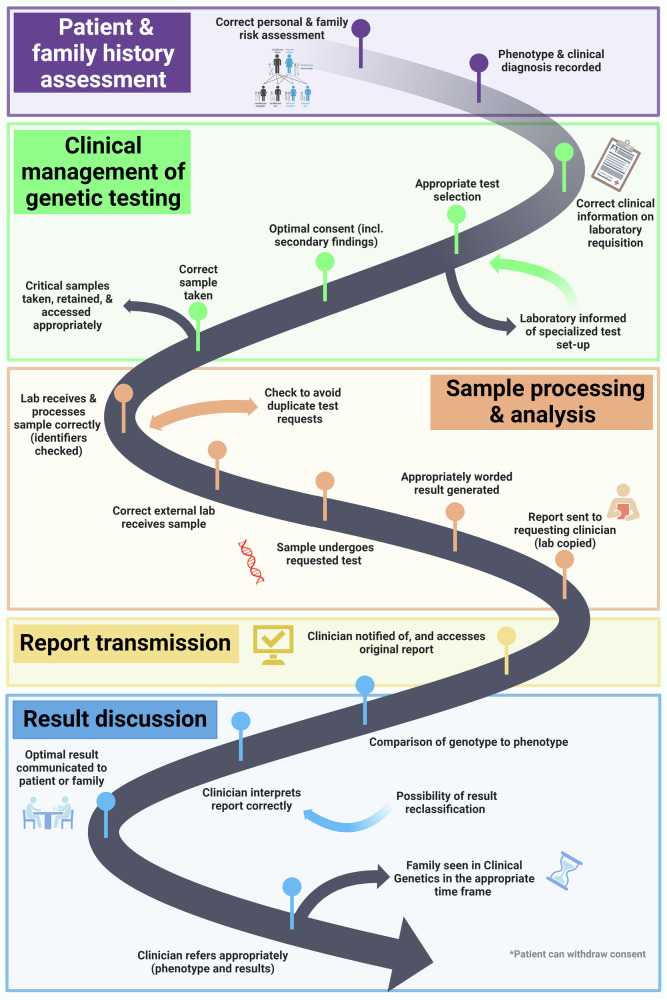
Process map of the Clinical Genetics Patient Journey. The process map describes the 22 key steps where risk occurs if care is not optimally managed. The map spans 5 sections of clinical and laboratory activity.

The process map developed from analysis of the cases has 22 steps spanning five stages: (1) patient and family history assessment (2) clinical management of genetic testing (3) sample processing and analysis (4) result transmission (5) result discussion, as shown in Fig. 2.

The final adapted version of the ‘Harm to a person (Service User, Patient, Staff & Public)’ severity table is shown as part of the risk assessment tool adapted for clinical genetics use, encompassing all steps in the traffic light system (Supplemental Fig. 2).

Feedback was solicited on the process map and audit form from the 6 clinical teams. All teams found the wording of the process map clear and did not identify any redundant or missing steps. Centres with developed IT infrastructure translated the form into a computerized version for ease of data capture.

### Audit

The results of the audit of all centres are shown in Table 3. The number of appointments with risk events ranged from 0.8% to 20.3%. The percentage of appointments with risk, where there was a failure at more than one step occurred ranged from 20.0% to 54%. Oulu noted a few risks compared to all other centres, all observed at the start of the process map. Risk events occurring at the start were also common in Oxford. Events during the sample processing section were common in Dublin, Belfast, Oxford, and Newcastle. Transmission of reports were an issue in Dublin and Belfast. Adverse events were common during the results discussion period in Dublin, Belfast, and Oxford. In Dublin, most risk was attributable to long waiting lists, IT deficiencies, gaps in non-geneticists’ genomic knowledge resulting in sub-optimal consent or misinterpretation of test reports, and wrong testing being ordered by mainstream clinicians due to the limited access to clinical genetic and laboratory expertise.

**Table 3:** Between-centre comparison of frequency of risk events recorded over 6-week prospective audit.

### Re-audit

Two units, Dublin and Oxford, were re-audited in 2024. In Dublin, 57 appointments of 671 had risk events (8.5%). In Oxford, 42 appointments of 1448 had risk events (2.9%), a 55% reduction.

## Discussion

The 22-step process map of the patient journey through clinical genetics is novel. Donohue et al. [7], whose qualitative survey examined a shorter journey surrounding genetic testing, identified 6 of the same steps: correct test ordered, communication between laboratory and provider; clear wording of report; correct interpretation; genotype to phenotype correlation; and clear explanation of testing results. Although Donohue et al. [7] did not order the steps, they cited them all as contributing factors to the misinterpretation of genetic testing with resulting patient impact.

While all centres that took part in the research identified adverse events, some of the variances noted can be explained by differing practices and staff roles. The difference in percentage of appointments with risk events identified between centres may relate to where in the pathway those staff completing forms are active. The reduced capture of laboratory events by Dublin, Belfast, Craiova, and Oulu was noted, where clinical teams used external laboratories, leading to less engagement with laboratory processes.

Despite not capturing laboratory events, 1 in 10 appointments in Dublin had an adverse event, and nearly half of those had risk at more than one step in the pathway. The majority of risk was attributable to: (1) long waiting lists, (leading to a pregnancy occurring whilst waiting for existing child to be seen, death of proband on the waiting list or critical sample being discarded before appointment), (2) IT deficiencies, (poor inter-operability between hospitals resulting in convoluted processes to request tests by mainstream and clinical genetics clinicians (adding time to the already limited time to review and discuss patients) test reports not being accessible or tests being duplicated unnecessarily (3) insufficient access to clinical genetics expertise, for example, to support mainstream clinician with queries regarding complicated test reports such as imprinting, resulting in mis-information being given to the family by the mainstream team, (4) gaps in non-geneticists’ genomic knowledge including informed consent when testing, resulting in secondary findings being reported and the family not realising they had consented to this.

Whilst some of these critical steps could be specific to Dublin, as for the example the lack of a test directory, (present in England), others risks are shared within most centres, such as lack of sufficient specialist staff to support mainstream clinicians in the early steps of testing and limited clinical genetics time to support mainstream colleagues and the limitations due to poor interoperability of IT systems.

In Craiova, only one clinician recorded risks, and these were noted throughout the patient journey. All other centres recorded adverse events by multiple team members. The small numbers of adverse events recorded in Newcastle were thought to reflect staff time pressures precluding filling in the audit form. In Dublin, the Principal Investigator (SAL) captured and logged adverse events raised by any staff members at clinical meetings. Belfast suggested under-reporting of adverse events, and a high percentage identified with multiple risks due to staff only recognizing risk when > 1 step failed across the process map. The high percentage of declared risk events in all centres where more than 1 step failed suggests the possibility that staff are only recognizing or recording appointments where the most severe harm occurs.

Oxford recorded many events at sample receipt, but they have a dedicated genomic practitioner team whose goal is to facilitate the consenting process and complete and collate paperwork and samples. Oxford has recognized the need for a quality check process at receipt of sample, allowing any concerns regarding consent to be flagged, demographic errors to be fixed, and allowing clinical questions to be managed with the referring physician early in the testing process, ensuring the correct test is done for genetic tests requested by mainstream teams. Although Oxford recorded many of these sample receipt events, the presence of the genomic practitioner corrected the event so that there were fewer events downstream in the pathway. No other participating centre has this system in place, although a recent review describes the implementation of genetic counsellors into genomic test ordering review as both cost-saving and beneficial to patients [27]. Some adverse events noted by Oxford may have been picked up within laboratory systems in the other units and not have been recorded by the clinical teams elsewhere.

Oulu identified only five events over 6 weeks. In comparison to the other 5 centres, they have limited involvement of mainstream clinicians in genetic testing. For straightforward referrals, they have developed lean processes where genetic nurses have a central role, coordinating the majority of genetic testing, including consent and test requisitions. Interestingly, all events in Oulu occurred early in the clinical pathway suggesting the genetic nurses are capturing events early on and reducing the number of adverse events later in the pathway. Most of the other centres do not have these same pre-test control systems in place, apart from laboratory scientist gatekeeping. It suggests that some downstream adverse events occurring in Dublin, Belfast, Newcastle, and Craiova may result from a lack of early pathway controls.

Following the first audit, Dublin adjusted triage priorities to ensure referrals with critical samples were seen promptly, and the team educated mainstream clinicians regarding European guidelines on secondary findings [28], and a reduction in these referrals was noted on re-audit. The number of adverse events stayed static in Dublin, although the severity of the risk was reduced (e.g., no loss of critical samples or referrals for secondary findings were noted on repeat audit). Between audit and re-audit, Oxford introduced proactive training and education of mainstream clinicians, encouraging the pathway for Genomic Practitioners to complete the records of the discussion with the patients. These initiatives resulted in a reduction in adverse events, particularly at the receipt of the sample. This change of process may explain the reduction in adverse events noted on re-audit. However, the repeat audit in Oxford noted reduced engagement by staff in audit form completion, which may also have been a factor.

Risk reducing practices that could be implemented in other centres to help reduce risk include: (1) specialised staff to support early steps in the patient pathway, ensuring optimum consent etc, (2) robust IT infrastructure and connectivity ensuring timely access to reports, and robust IT infrastructure and administrative support to plan and record Multi-Disciplinary Teams outcomes (3) a genetic testing directory and adequate resource for laboratory staff to facilitate clinical and laboratory gatekeeping, (4) sufficient dedicated clinical genetics time in their job plans to address mainstream clinicians’ enquiries and MDTs for complex results to support colleagues with interpretation and communication of complex genomic results.

One participant noted that, when working in a high-risk environment, staff may become complacent about adverse events. Complacency with risk does not make the patient environment safe. Risk management departments encourage staff to conduct risk assessments and report incidents; however, over-reporting of adverse events can lead to ineffective feedback limiting the ability for risk mitigation and improvement [29]. This can leave staff demoralised and further decrease the likelihood of effective risk reporting and resolution. Our audit tools may allow centres to prospectively identify systematic risk and target the most severe risk areas pertinent to their centre for risk mitigation to improve quality, rather than wait for adverse events to occur and report them retrospectively.

Shebl et al. [30] discuss four types of validity with respect to risk assessment tools (face, content, criterion, and construct validity). We have established face validity – an independent, subjective assessment of whether the tools meet their stated aim – by the opinion of the participating clinical teams and risk experts. Content validity – whether the tools contain the domains they intend to measure – was confirmed by independent clinical teams testing to audit tool and providing feedback about the process map and the audit’s ability to capture perceived risk in their clinical service. The content validity of the impact table was confirmed as many of the themes of ‘harm to a patient’ identified by the research team mirror those identified in our patient survey [26], and align with perceived consequences of errors (incorrect diagnosis, incorrect treatment, stress for patient and family, and ethical concerns) described by Donohoe et al. [7]. Criterion validity could not be assessed due to the lack of a comparable measure, as hospital clinical and laboratory risk registers capture adverse events but not near-miss data. Construct validity, whether the measurement tool (risk matrix) measures the concept of the magnitude of risk, was not assessed. However, the integration of HSE’s traffic-light risk assessment tools [23] increases acceptance of the measure and competence in its use.

Once published, future consideration will be given to the use of the risk assessment tools in the Republic of Ireland and in the South-West of Britain.

A limitation of this study is the possibility that the process map is specific to European clinical genetic processes. Expansion of this research would be to trial these tools in private clinical genetics, clinical genetics outside of Europe, and mainstream clinical teams where genetic testing is routinely used. Reproducibility of the genetics-specific risk assessment severity matrix could be demonstrated by a trial with a different dataset of cases with risk identified would be warranted.

In conclusion, each discipline understands its own specialty-specific adverse events. By developing specialty-specific risk assessment tools and reminding staff to log adverse events, it becomes clearer where in the patient journey risk is occurring and where action is needed. We propose that this is a robust risk assessment tool that should allow specific risk areas to be identified and result in a more focused approach to risk mitigation. It provides a framework to help healthcare providers outside of genetics appreciate all the steps that are necessary for safe patient care. This process map and risk assessment tool may (1) allow genetic teams to prospectively identify risk areas in their practice and put controls in place if system errors are identified (2) be used to investigate serious adverse events that require a retrospective review, (3) be used as an investigative tool to manage patient complaints and (4) teach clinical staff about the steps needed for safe and ethical genetic testing. Introduction of this tool on a rolling basis could allow the evaluation of risk mitigation strategy implementation as part of a quality management system.

## Supplementary information

Supplemental Figure 1

Supplemental Figure 2

## Data Availability

The data that support the findings of this study are not openly available due to the possibility of identification, as cases reviewed were families with rare diseases. These are available from the corresponding author upon reasonable request. Data are located in controlled access data storage at University College Dublin.
