# Supplementary material for: What is risk in clinical genetics? Designing and piloting tools to evaluate risk in clinical genetics using failure modes and effects analysis
Source: Eur J Hum Genet. 2025 Oct 27;34(4):505–14. doi: 10.1038/s41431-025-01961-3 (PMC13047049; doi:10.1038/s41431-025-01961-3)
Supplement: Supplementary file 1 — Supplemental Figure 1 [file 41431_2025_1961_MOESM1_ESM.pptx]

## Slide 1
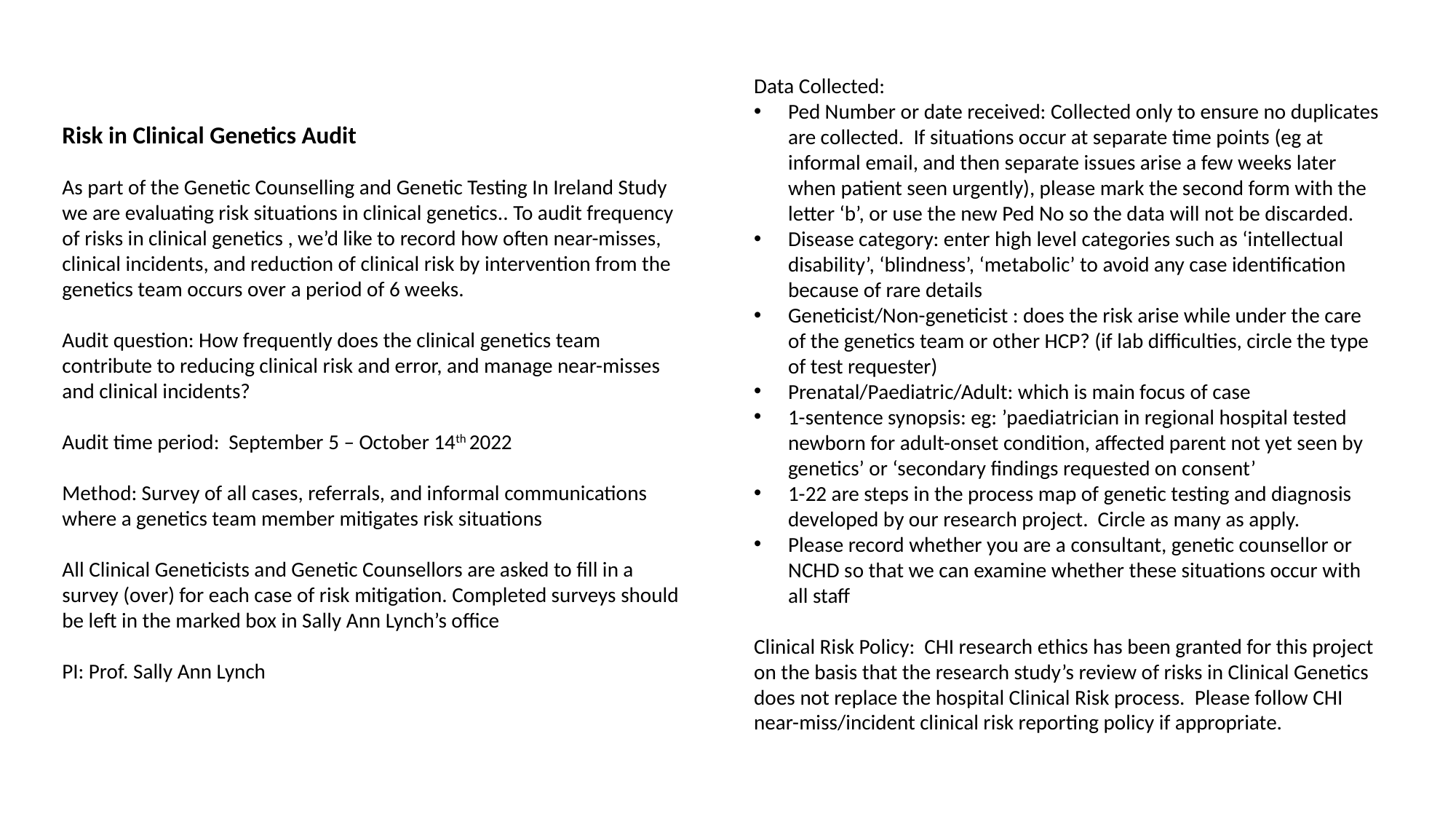

Data Collected:
Ped Number or date received: Collected only to ensure no duplicates are collected. If situations occur at separate time points (eg at informal email, and then separate issues arise a few weeks later when patient seen urgently), please mark the second form with the letter ‘b’, or use the new Ped No so the data will not be discarded.
Disease category: enter high level categories such as ‘intellectual disability’, ‘blindness’, ‘metabolic’ to avoid any case identification because of rare details
Geneticist/Non-geneticist : does the risk arise while under the care of the genetics team or other HCP? (if lab difficulties, circle the type of test requester)
Prenatal/Paediatric/Adult: which is main focus of case
1-sentence synopsis: eg: ’paediatrician in regional hospital tested newborn for adult-onset condition, affected parent not yet seen by genetics’ or ‘secondary findings requested on consent’
1-22 are steps in the process map of genetic testing and diagnosis developed by our research project. Circle as many as apply.
Please record whether you are a consultant, genetic counsellor or NCHD so that we can examine whether these situations occur with all staff
Clinical Risk Policy: CHI research ethics has been granted for this project on the basis that the research study’s review of risks in Clinical Genetics does not replace the hospital Clinical Risk process. Please follow CHI near-miss/incident clinical risk reporting policy if appropriate.
Risk in Clinical Genetics Audit
As part of the Genetic Counselling and Genetic Testing In Ireland Study we are evaluating risk situations in clinical genetics.. To audit frequency of risks in clinical genetics , we’d like to record how often near-misses, clinical incidents, and reduction of clinical risk by intervention from the genetics team occurs over a period of 6 weeks.
Audit question: How frequently does the clinical genetics team contribute to reducing clinical risk and error, and manage near-misses and clinical incidents?
Audit time period: September 5 – October 14th 2022
Method: Survey of all cases, referrals, and informal communications where a genetics team member mitigates risk situations
All Clinical Geneticists and Genetic Counsellors are asked to fill in a survey (over) for each case of risk mitigation. Completed surveys should be left in the marked box in Sally Ann Lynch’s office
PI: Prof. Sally Ann Lynch

## Slide 2
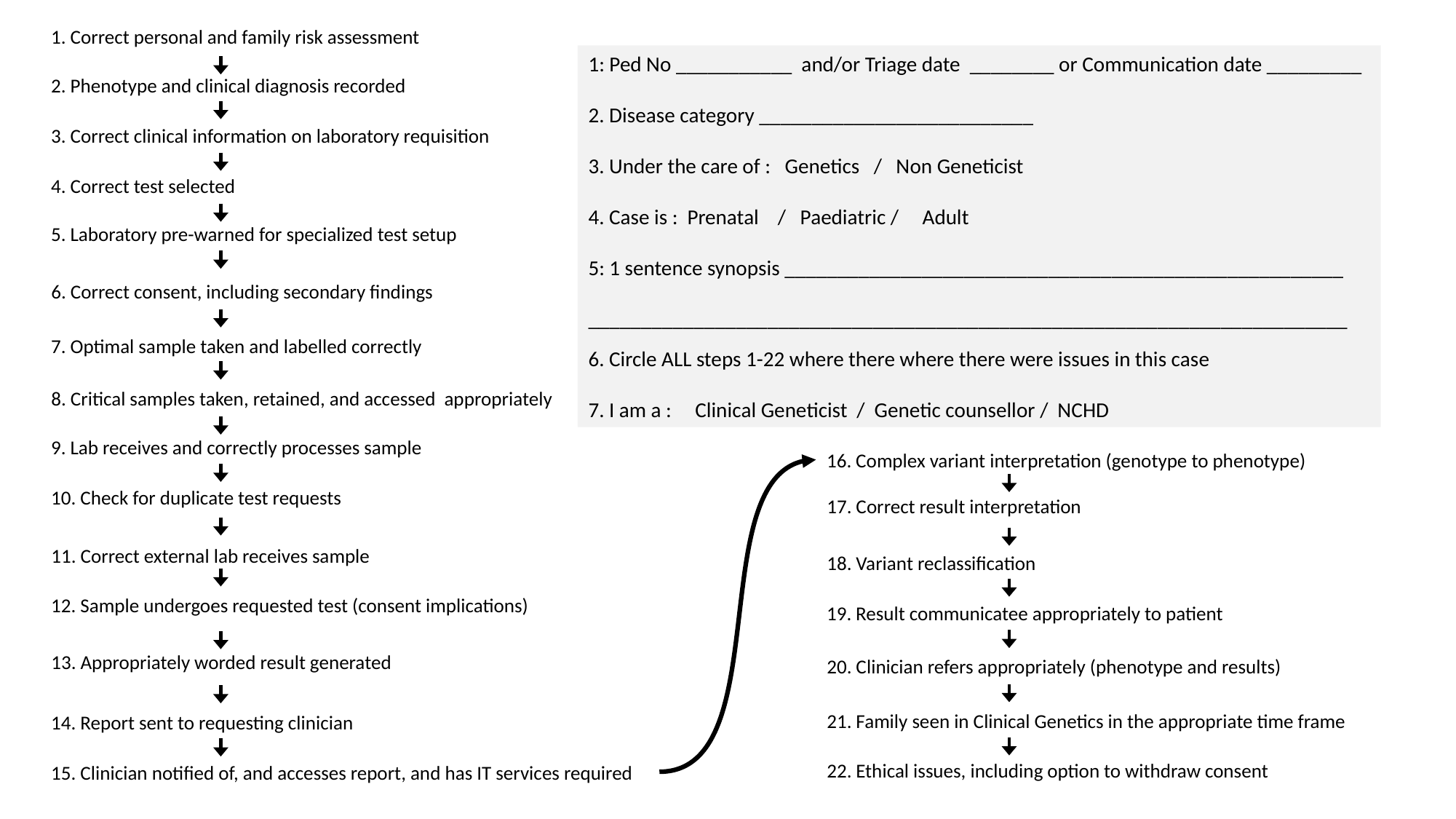

1. Correct personal and family risk assessment
1: Ped No ___________ and/or Triage date ________ or Communication date _________
2. Disease category __________________________
3. Under the care of : Genetics / Non Geneticist
4. Case is : Prenatal / Paediatric / Adult
5: 1 sentence synopsis _____________________________________________________
________________________________________________________________________
6. Circle ALL steps 1-22 where there where there were issues in this case
7. I am a : Clinical Geneticist / Genetic counsellor / NCHD
2. Phenotype and clinical diagnosis recorded
3. Correct clinical information on laboratory requisition
4. Correct test selected
5. Laboratory pre-warned for specialized test setup
6. Correct consent, including secondary findings
7. Optimal sample taken and labelled correctly
8. Critical samples taken, retained, and accessed appropriately
9. Lab receives and correctly processes sample
16. Complex variant interpretation (genotype to phenotype)
10. Check for duplicate test requests
17. Correct result interpretation
11. Correct external lab receives sample
18. Variant reclassification
12. Sample undergoes requested test (consent implications)
19. Result communicatee appropriately to patient
13. Appropriately worded result generated
20. Clinician refers appropriately (phenotype and results)
21. Family seen in Clinical Genetics in the appropriate time frame
14. Report sent to requesting clinician
22. Ethical issues, including option to withdraw consent
15. Clinician notified of, and accesses report, and has IT services required
