# Supplementary material for: What is risk in clinical genetics? Designing and piloting tools to evaluate risk in clinical genetics using failure modes and effects analysis
Source: Eur J Hum Genet. 2025 Oct 27;34(4):505–14. doi: 10.1038/s41431-025-01961-3 (PMC13047049; doi:10.1038/s41431-025-01961-3)
Supplement: Supplementary file 2 — Supplemental Figure 2 [file 41431_2025_1961_MOESM2_ESM.docx]

**Clinical Genetics Risk Assessment Tool**

- Step 1. Pinpoint the risk event on the clinical genetics process map. One or more missteps may happen in a single event. If more than one process map step has a risk identified, mark the primary or root cause risk in the first column.

| **Clinical Genetics Clinical Process Map and Missteps Along the Map** | | |
| --- | --- | --- |
| **Is this the primary step broken? (Y/N)** | **Process map steps** | **Missteps along the map** |
|  | 1. Family and risk assessment | Incomplete or incorrect assessment |
|  | 2. Phenotype and clinical diagnosis recorded | Ubiquitous sign not reported, hard sign not reported, phenotype poorly recorded in referral letter |
|  | 3. Clinical information on lab requisition | Incomplete or incorrect clinical information (phenotype) on lab requisition |
|  | 4. Appropriate test selection | Suboptimal / Inappropriate test selection. Correct test delayed or not done |
|  | 5. Lab informed of specialized test set up | Laboratory not informed of specialized test requirement. Test not available in time frame required. |
|  | 6. Patient consented (and with respect to secondary findings) and consent documented as per institution and professional guidelines^1^ | Inadequate Consent OR Minor consented inappropriately for adult-onset conditions OR HCP taking consent does not understand test ordered, not complying with institution and professional guidelines^1^ |
|  | 7. Correct sample taken and labelled in accordance with hospital and laboratory procedures / guidelines | Sample not taken properly (incorrect or suboptimal sample type, incorrect tube, incorrect medium, suboptimal or incorrect transport conditions) and labelled in accordance with hospital and laboratory procedures / guidelines |
|  | 8. Critical sample taken, retained and accessed appropriately | Critical sample (prenatal sample, newborn sample, critically ill patient, pre-transfusion sample) not taken or not retained |
|  | 9. Lab received sample and processes correctly | Sample not received by laboratory, sample not received in correct time frame, sample not processed correctly |
|  | 10. Duplicate sample check | No check for duplicate sample, sample accepted for analysis without a check |
|  | 11. Sample sent to correct external lab | Sample sent to incorrect or suboptimal external lab |
|  | 12. Sample undergoes requested test | Sample does not undergo requested test in appropriate time frame, or undergoes sub-optimal or incorrect test |
|  | 13. Appropriately worded result generated | Report sent out with confusing wording, incorrect result, report sent with insufficient or incorrect interpretation. Report transcribed by secondary processor. |
|  | 14. Report sent to requesting clinician in accordance with National Hospital Laboratory Accreditation regulations, laboratory policies, and GDPR and data protection policies | Report not sent to requesting clinician in accordance with National Hospital Laboratory Accreditation regulations, laboratory policies, and GDPR and data protection policies |
|  | 15. Clinician notified of and can access test report | Clinician not notified of and/or cannot access report due to errors in report sending, suboptimal ICT systems |
|  | 16. Genotype to phenotype comparison | Incorrect genotype to phenotype comparison (consider whether genotype is causal/pathogenic with respect to disease or an incidental finding) |
|  | 17. Clinician correctly interprets report | Clinician doesn’t read and interpret report properly. Does not correctly interpret for at-risk relatives as well as person tested |
|  | 18. Variant reinterpretation | Pathogenicity of genetic variants was not re-evaulated periodically to see if classification changed |
|  | 19. Result communicated clearly to family | Result and implications not communicated clearly to family by clinician team who ordered the test OR result and its personal and relatives’ implications not communicated clearly (next steps for at-risk family members identified) |
|  | 20. Clinician refers to genetics appropriately | Clinician does not refer to genetics OR refers with insufficient or incorrect information OR does not offer referral for appropriate clinical follow-up |
|  | 21. Seen in the appropriate time frame in genetics | Not seen in the appropriate time frame in genetics, or not triaged appropriately or inappropriate rejection from service or prioritisation on waiting list |
|  | 22. Ethics | No mechanism for patient to withdraw consent, or other |

^1^ de Wert, G., Dondorp, W., Clarke, A. *et al.* Opportunistic genomic screening. Recommendations of the European Society of Human Genetics. *Eur J Hum Genet* **29**, 365–377 (2021). [https://doi.org/10.1038/s41431-020-00758-w date accessed July 23](https://doi.org/10.1038/s41431-020-00758-w%20date%20accessed%20July%2023), 2025.

2. Evaluate the magnitude of the risk in the event using:

- The table below for different harms to a patient and their family in clinical genetics
- The HSE Risk Assessment tool for all categories of risk except ‘Harm to a Person’ <https://www.hse.ie/eng/about/who/riskmanagement/risk-management-documentation/hse-enterprise-risk-management-supporting-tools/hse-risk-assessment-tool.pdf>

|  | | **Harm Severity Score** | | | | |  |
| --- | --- | --- | --- | --- | --- | --- | --- |
| **Possible harms** | | **1** | **2** | **3** | **4** | **5** |  |
|  | delayed / missed / suboptimal / inappropriate surveillance, management (incl surgery), pharmaceutical intervention opportunities for proband or family mber | Delayed or suboptimal treatment leads to no harm | Lack of opportunity for treatment or management leads short term, minor harm such as lessening of bodily, sensory, motor, physiologic or intellectual function | Lack of opportunity for treatment or management leads to moderate (significant but not permanent) harm such as permanent lessening of bodily, sensory, motor, physiologic or intellectual function | Lack of opportunity for treatment or management leads to severe harm such as permanent lessening of bodily, sensory, motor, physiologic or intellectual function resulting in long-term incapacity or disability | Lack of opportunity for treatment or management leads to death or permanent disability in person or family member |  |
|  | delayed / missed / suboptimal / inappropriate reproductive options, prenatal testing opportunities for proband or family member | All reproductive options and/or prenatal testing known pre-conceptually | All reproductive options and/or prenatal testing available but only during a pregnancy | Limited/suboptimal reproductive options and/or prenatal testing available | Wrong testing offered or no reproductive options or prenatal testing available in time frame: no occurrence or recurrence of the condition | Wrong testing offered or no reproductive options or prenatal testing available in time frame: leading to occurrence/recurrence in person or family member |  |
|  | Psychosocial functioning (anxiety, depression, etc) including impact on major life decisions (childbearing, employment, education, relationships) in person or family members, or lack of access to support community. | No impaired psychosocial functioning | Impaired psychosocial functioning >72hrs to less than 1 month | Impaired psychosocial functioning over 1 month - less than 6 months | Impaired psychosocial functioning over 6 months | Permanent psychosocial functioning incapacity. Major life decisions (childbearing, exployment, education, relationships) impacted in person or family members. |  |
|  | Personal or family financial harm (loss of earnings, health insurance, life assurance, mortgage) or out of pocket medical expenses | Financial loss causing no hardship | Short term financial loss causing minor hardship | Temporary financial loss causing moderate hardship | Financial loss causing sustained or major hardship, causing long term consequences | Financial loss causing extreme or permanent hardship |  |

3. Using table below, score how likely a break is likely occur in the (primary) step you have identified in #1.

**
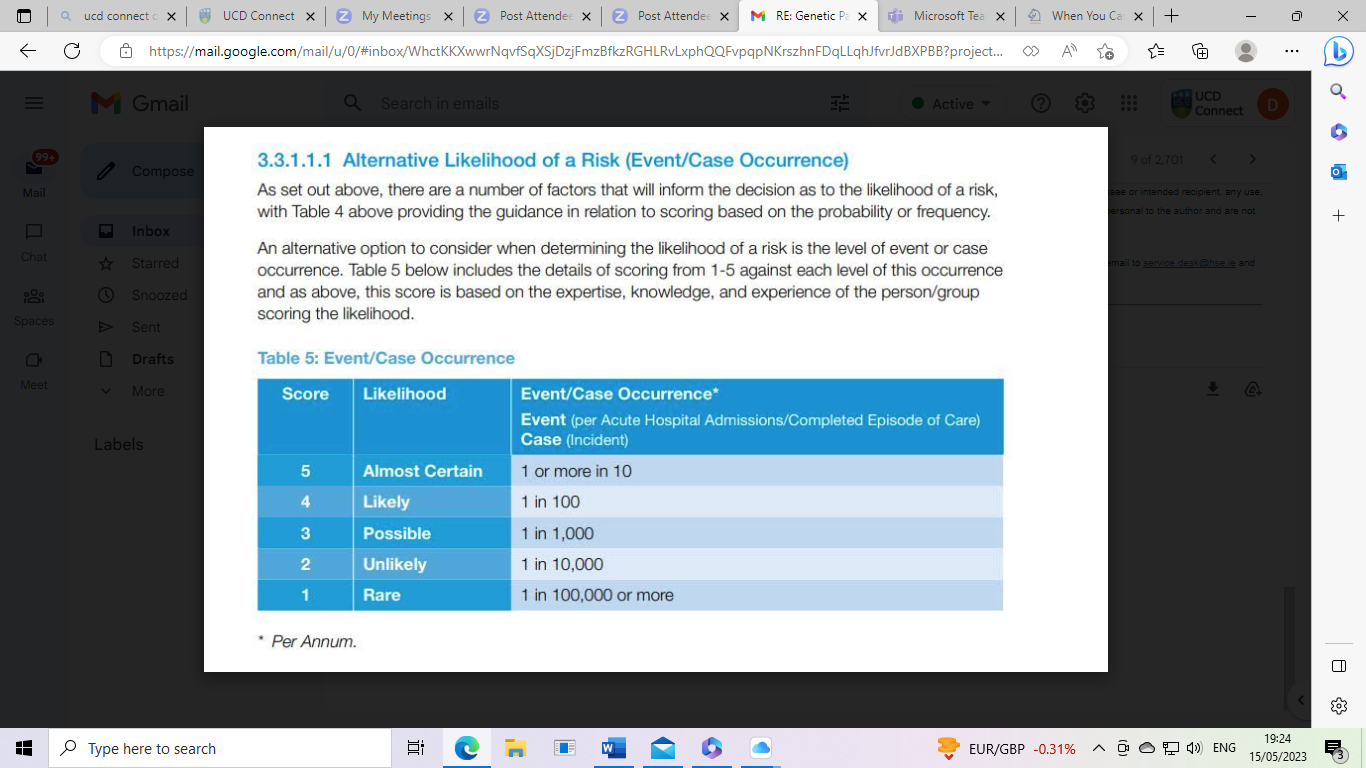
**

4. Multiply the impact score in the primary pathway step broken by the event score to get a number 1 - 25. When more than 1 type of harm occurs, use the highest severity of harm identified. Colour code the risk from the following table:


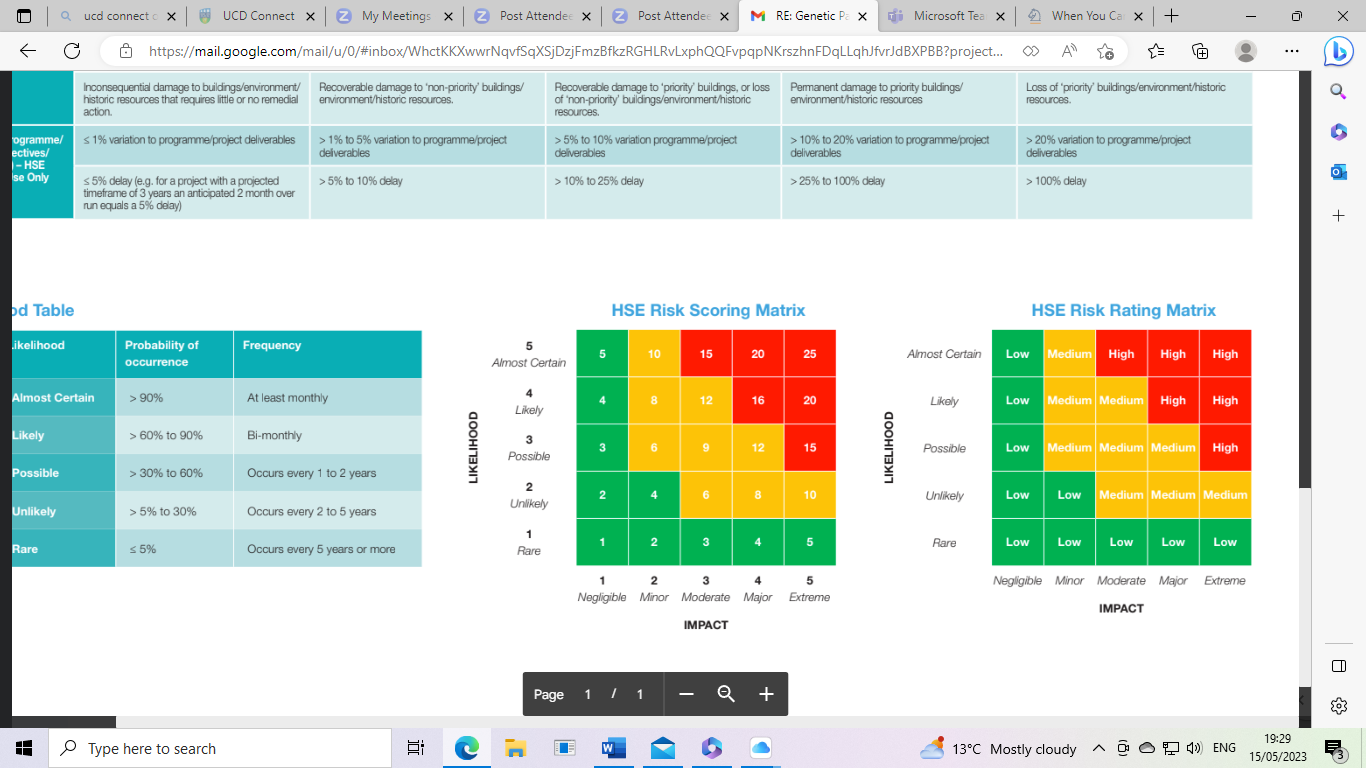


Steps 3 and 4 taken from 2023 HSE Enterprise Risk Management Supporting Tools (<https://www.hse.ie/eng/about/who/riskmanagement/risk-management-documentation/hse-enterprise-risk-management-supporting-tools/>). Please refer to this site for additional information about the adverse event severity scoring.
